# Enzootic Circulation, Massive Gull Mortality and Poultry Outbreaks during the 2022/2023 High-Pathogenicity Avian Influenza H5N1 Season in the Czech Republic

**DOI:** 10.3390/v16020221

**Published:** 2024-01-31

**Authors:** Alexander Nagy, Martina Stará, Lenka Černíková, Eliška Kličková, Ondřej Horák, Lada Hofmannová, Kamil Sedlák

**Affiliations:** State Veterinary Institute Prague, Sídlištní 136/24, 165 03 Prague, Czech Republic; martina.stara@svupraha.cz (M.S.); lenka.cernikova@svupraha.cz (L.Č.); eliska.klickova@svupraha.cz (E.K.); ondrej.horak@svupraha.cz (O.H.); lada.hofmannova@svupraha.cz (L.H.); kamil.sedlak@svupraha.cz (K.S.)

**Keywords:** H5N1, HPAI, avian influenza, high-pathogenicity avian influenza, outbreak, poultry, genotype, AB, AF, CH, BB, influenza in gulls

## Abstract

In 2022/2023, Europe experienced its third consecutive season of high-pathogenicity avian influenza. During this period, the Czech Republic was again severely affected. For the first time, the number of culled birds approached one million, which was three times higher than in previous seasons. In parallel to the outbreaks in poultry, mass die-offs of gulls were also observed. In the present study, we performed whole-genome sequencing and phylogenetic analysis of 137 H5N1 strains collected in the Czech Republic in 2022/2023 (94.6% of all outbreaks or locations). The analysis revealed four distinct genotypes: AB, CH, BB and AF. Phylogenetic analysis suggested that the AF genotype persisted from the previous H5N1 season without reassortment. In addition, the genotype BB, which was detected mainly in gulls, showed a noticeable strain diversity at the local level. This virus was also responsible for a single outbreak in commercially bred turkeys. Finally, an interesting spatio-temporal cluster with three co-circulating H5N1 genotypes, AB, CH and AF, was identified with no evidence of intrasubtype reassortment. Highly sensitive molecular surveillance and the timely sharing of genomic sequences and associated metadata could greatly assist in tracking the spread and detecting molecular changes associated with the increased virulence of this potentially zoonotic pathogen.

## 1. Introduction

High-pathogenicity avian influenza (HPAI) is an extremely contagious, multiorgan systemic disease of poultry that causes high mortality and significant economic losses. Since October 2021, Europe has been affected by HPAI with a predominance of the H5N1 subtype and with the H5 hemagglutinin (HA) belonging to subclade 2.3.4.4b. In 2021/2022, the H5N1 HPAI viruses also caused the largest epidemic ever observed in Europe [1]. The 2021/2022 epidemic is significant not only because of its unprecedented geographical spread and the number of wild birds and poultry infected but also because, for the first time, two main sources of infection were identified. 

Until 2021/2022, a seasonal pattern of H5Nx HPAI incidence was observed in Europe [2]. As with low-pathogenicity avian influenza in mild temperate zones [3], an HPAI season started in autumn and was seeded by multiple incursions of H5Nx HPAI strains circulating outside Europe, showing a wide geographical distribution from Africa and the Middle East to Russia [2,4,5,6]. The resurgent strains then spread throughout Europe and circulated until the spring of the following year. However, from April or May onwards, the incidence of HPAI detections usually began to decline, followed by a latency period in the summer during which HPAI strains disappeared or were detected very sporadically [7,8]. Due to the consistent seasonality observed in European outbreaks, an official HPAI season has been defined for reporting purposes, beginning from October (week 40) and lasting until the end of September (week 39) of the following year [9].

However, during the summer of 2021, an unusually high number of H5N1 HPAI infections were detected in Europe. Despite a sharp decline in incidence, as in previous years, the H5N1 HPAI virus was reported in wild waterfowl in northern Europe [10]. In addition, mass mortality was reported in great skuas (*Stercorarius scua*) on several islands of the Scottish mainland [11]. As a result, phylogenetic analysis since October 2021 revealed a new source for the 2021/2022 season, parallel to the resurgent strains [6]. This was represented by resident enzootic H5N1 HPAI viruses that persisted in the northern European wild bird population during the summer of 2021.

However, in the following summer of 2022, an even greater persistence of H5N1 HPAI was observed. As a result, catastrophic adult and chick mortality, estimated at 70–100% in some breeding colonies, was reported in many parts of northern Europe, often in remote areas. Among the most severely affected wild bird species were sandwich terns (*Thalasseus sandvicensis*), common terns (*Sterna hirundo*), northern gannets (*Morus bassanus*) and, again, great skuas [12,13,14,15,16]. In addition, some populations were already decimated in 2021 [15]. In terms of H5N1 HPAI ecology, seabirds appear to have served as an alternative ecological niche, allowing the virus to persist in Europe throughout the summer of 2022. From there, infections spread to black-headed gulls and back to wild waterfowl in the autumn of 2022 [17], facilitating the spread of the virus further inland and thus igniting the 2022/2023 season.

Although the full ecological impact of the massive 2022 seabird die-off will only become apparent in the coming years, the summer persistence supports a previously observed shift in the infection dynamics of H5N1 HPAI in Europe [6]. The enzootic nature of the disease poses a new threat to both wild birds and domestic poultry. In particular, the resident strains may be an important parallel or even the sole source of the next epidemic. Accordingly, available genetic data up to March 2023 reported the circulation of resident European strains without any detected virus incursions into Europe [17].

In 2022/2023, the H5N1 HPAI outbreak in Europe also had a significant impact on the poultry industry in the Czech Republic. Different H5N1 genotypes and massive infections in gulls were identified. In this article, we investigate the origin, geographic distribution and genotype diversity of the H5N1 viruses detected in the Czech Republic during the 2022/2023 HPAI season. To this end, whole-genome sequencing, spatio-temporal and phylogenetic analyses of 137 H5N1 HPAI strains collected from wild birds and backyard and commercial poultry were performed.

## 2. Materials and Methods

All bird carcasses received were necropsied. Cloacal or tracheal swabs or parts of multiple organs were collected for virus detection and next-generation sequencing. Swabs were vortexed in PBS, and the pooled organs were homogenized in RNA later solution (Invitrogen, CA, USA). Total nucleic acid was extracted from 200 µL of supernatants of pooled organs and swabs (MagNAPure Compact, MagNAPure 24 or MagNAPure 96 instruments, Roche) and eluted into 50 µL. RT-qPCR methods (specific for generic influenza A virus, H5 and N1 subtypes) and the 2.3.4.4b H5 HA cleavage site [18,19,20,21] were used for the detection, identification and pathotyping of H5N1.

Real-time next-generation sequencing was performed using the Nanopore technology (MinION Mk1B, R10.4.1 flow cells; Native Barcoding Kit (SQK-LSK-114.96), (Oxford Nanopore Technologies, Oxford, UK)). The H5N1 genome was reverse transcribed (SuperScript III and SuperScript IV, ThermoFisher Scientific, MA, USA) using the Uni12 forward primer and amplified in a set of three PCR reactions (Q5 HiFi DNA polymerase, New England Biolabs; primers available on request). The PCR reactions were pooled, and the resulting sequencing libraries were purified (SPRIselect beads; Beckman Coulter, CA, USA) and quantified (QIAxpert; Qiagen, Hilden, Germany). End preparation, native barcoding and sequencing adapter ligation were performed according to the manufacturer’s instructions. Sequencing runs were operated via MinKNOW standalone software (v2.3.04.3 or higher) with a sequencing speed of 400 bps and excluding reads ≤ 200 nucleotides. Basecalling was performed with Guppy (6.4.2 and higher) using the super accurate basecalling model (dna_r10.4.1_e8.2_400 bps_sup.cfg config file). The run was monitored in real-time by implementing the RAMPART (Read Assignment, Mapping and Phylogenetic Analysis in Real Time) module of the ARTIC bioinformatic pipeline [22] set to the concatenated H5N1 genome as a reference. Demultiplexing adapter and barcode trimming were performed using Porechop and Guppy. Consensus sequences were obtained using the Bcftools mpileup [23], NGSpeciesID [24] and Amplicon sorter [25] tools. The consensus sequences were aligned using MAFFT (Multiple Alignment using Fast Fourier Transform) [26] and manually curated to obtain a final consensus genome. Finally, the obtained genomes were annotated using the influenza virus sequence annotation tool [27]. All H5N1 genomes were submitted to the GISAID EpiFlu database with accession codes listed in Supplementary Table S1. The Czech H5N1 genomes were compared with other European sequences collected between 2022 and 2023 (GISAID database). Sequences were aligned using MAFFT, with alignment trimming and format conversion (Phylip full names and padded) using AliView [28]. Maximum likelihood (ML) trees (IQ-TREE multicore version 2.2.0-beta for Linux 64-bit [29]; 1000 replicates) were calculated separately for each genomic segment and as a concatenated genomic tree. Sequence concatenation was performed using the Union programme from EMBOSS [30] in the following order: PB2, PB1, PA, H5, NP, N1, MP and NS. For all trees, the best-fitting model was selected according to the Bayesian information criterion.

## 3. Results

### 3.1. Overview of the 2022/2023 H5N1 HPAI Season in the Czech Republic

**Figure 1:**
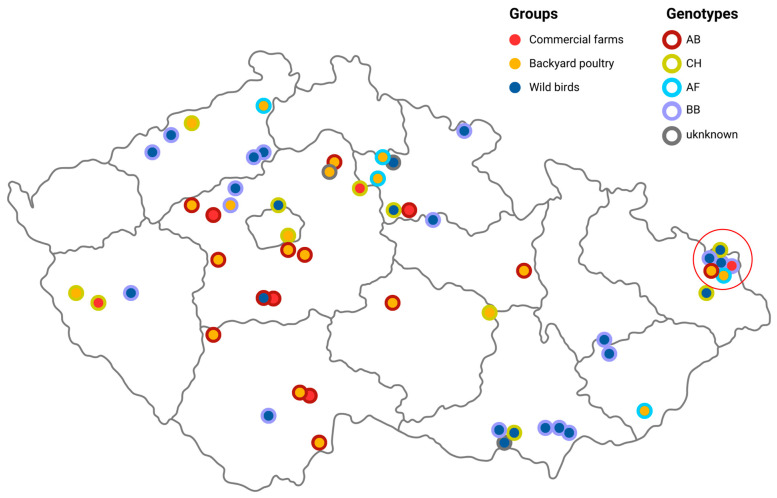
Geographical distribution of the H5N1 2022/2023 outbreaks and their corresponding genotypes on a blind map of the Czech Republic. Solid dots indicate bird categories (wild, backyard and commercial). Genotype information is represented using a halo. A red circle indicates a spatio-temporal cluster (please refer to the text). An interactive version of the map categorized using bird category is available online at https://www.google.com/maps/d/u/1/edit?mid=1F2miwu6f3LwcERhDkZGK-ztunikfBg0&ll=49.757942391979654%2C15.579510149999987&z (accessed on 29 January 2024) and by genotype at: https://www.google.com/maps/d/viewer?mid=1TK7NNdEvvBuOskqEvAHU0KMn6RLhGAc&ll=49.929839528405346%2C15.65820290000002&z (accessed on 29 January 2024).

The Czech H5N1 HPAI 2022/2023 index case is dated 1 December 2022 on a commercial farm with ~22,000 29-day-old Pekin ducks (Frahelž, South Bohemia region; ID 22394/22; Supplementary Table S1. For the individual locations of the outbreaks, please refer to the interactive maps via the links in Figure 1). Within a few days, the mortality of the ducklings increased rapidly to ~7000. A commercial flock of ~120,000 chickens under the same ownership and located within a 10 km surveillance zone remained negative. Shortly after, additional H5N1 HPAI cases in backyard poultry scattered throughout the country started to accumulate, and on 30 December 2022, increased mortality was reported in a commercial flock of laying hens (Brod nad Tichou, Pilsen region; IDs 24009/22 and 58/23, 122-181/23, 872/23). The farm contained ~740,000 birds, representing 15% of all commercial hens in the Czech Republic, with a production capacity millions of eggs per week, and housed the birds in three two-storey breeding sheds 1–3. Each floor was divided into two sections, A and B, and each section contained two barns. The disease started with a locally elevated mortality (0.12%) observed in a single barn located on the second floor of the central shed (ID 24009/22). Egg production, drinking and feeding were normal. The samples from the remaining two sheds, 1 and 3, were RT-qPCR negative. Three days later, on 2 January 2023, the H5N1 HPAI virus was detected in section A of the same shed (ID 58/23). And finally, on 4 January 2023, increased mortality was observed in sheds 1 and 3 (IDs 178-181/23). As a result, the entire farm was depopulated, including the destruction of millions of eggs. This is the worst HPAI outbreak ever recorded in poultry in the Czech Republic.

During January 2023, further important outbreaks were reported in commercial turkeys and ducks (IDs 66/23, 636/23, 951/23 and 1398/23), accompanied by widespread infections in backyard poultry and sporadic cases in wild birds (mallards, swans, a goose and herons) scattered throughout the country. In addition, 13 surface swabs were collected from one of the affected barns of a commercial duck farm (ID 951/23) two days after the outbreak was identified. These included fan shovels, fences, internal and external door handles, gas pipe surfaces, tool handles, crates, entrance doors, fan grille, walls, feeders, drinkers, bucket handles and work shoes. All samples were positive, with Cq values ranging from 31 to 38 [18].

Another significant aspect of the 2022/2023 season was the massive mortality of gulls due to the H5N1 HPAI infection. Increased gull mortality was first reported on 4 April 2023 from two distant ponds (IDs 5152/23 and 5295/23). These were soon followed by reports from many different water bodies across the country, with tens to several hundred gull carcasses scattered on the pond banks or directly in the water. The spread in gulls was associated with the introduction of a novel H5N1 genotype, BB, which began to circulate widely in the EU in December 2022 [31]. This gull-adapted genotype was subsequently responsible for the outbreak in commercial turkeys on 2 May 2023 (IDs 6734/23, 7123/23 and 7124/23) and was transmitted to peregrine falcons (IDs 7459/23, 7735/23, 8420/23 and 10854/23) and common terns (ID 8807/23).

In total, during the 2022/2023 season, the H5N1 HPAI virus was responsible for seven outbreaks in commercial poultry (including chickens, ducks and turkeys) and 25 outbreaks in backyard poultry (including chickens, ducks, geese and guinea fowl). In parallel, the H5N1 HPAI virus was identified in wild bird carcasses collected in 25 localities (Figure 1). More than 845,000 poultry were destroyed as part of the outbreak management. No H5N1 infections in mammals or low-pathogenicity avian influenza viruses were detected.

### 3.2. Genotyping and Molecular Characterization of the Detected H5N1 Viruses

To capture the genetic diversity among the H5N1 HPAI strains detected in the Czech Republic during the 2022/2023 season, 137 viral genomes were sequenced and analysed. Whole-genome sequences were obtained from 7/7 (100%) commercial outbreaks, 24/25 (96%) from backyard poultry and 23/25 (92%) from wild birds. In total, genome sequences were available from 52/55 (94.5%) of outbreaks or locations. In addition, three H5N1 genomes were sequenced directly from environmental samples (IDs 1220/23) collected during the outbreak in commercial ducks (IDs 951/23).

Almost all the CZE 2022/2023 H5N1 strains contained the PLREKRRKR/GLF cleavage site motif corresponding to the HPAI phenotype. A minor HPAI motif PLRGKRRKR/GLF was observed in the genotype BB strains collected during the outbreak in commercial turkeys sampled several times between 2 and 5 May 2023 (IDs 6734/23, 7123/23 and 7124/23).

All Czech H5N1 strains contained mutations 123P, 133A, 154N, and 156A and IDs 22969 and 22910-22912/23 held the 210I change (A/Vietnam/1203/2004 (H5N1) H5 numbering) in the H5HA, which increase in vitro binding to “human-type receptors” [32]. The genotype BB viruses contained the mutation 52N in the NP protein, which promotes the zoonotic potential [33]. All these changes have occurred naturally in the European H5N1 viruses of avian origin [34].

**Figure 2:**
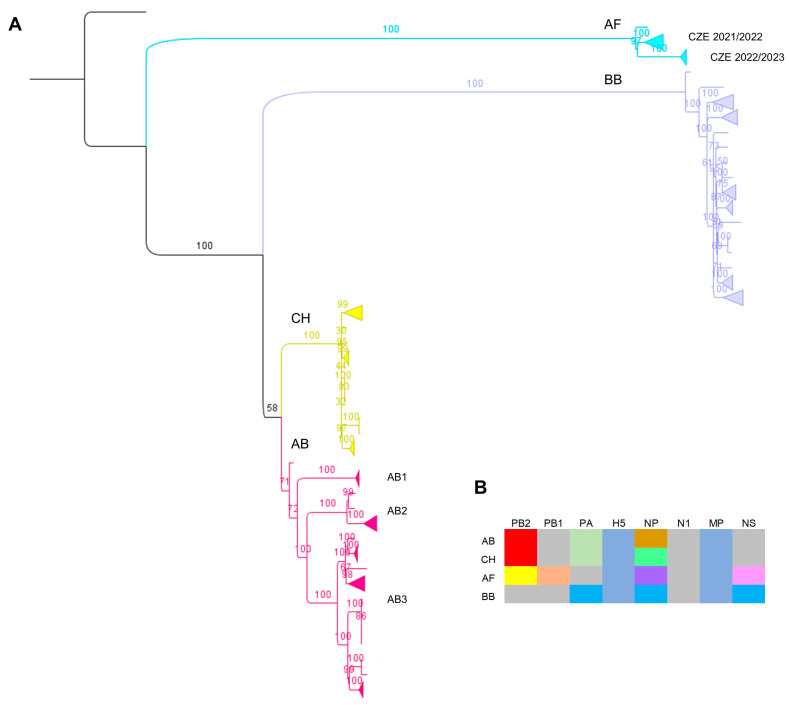
Genotyping of the Czech H5N1 strains detected during the 2022/2023 influenza season. In (**A**), the ML tree (GTR+F+G4 model) was calculated based on concatenated genomes together with the genotype reference strains and was rooted to A/Eurasian wigeon/Netherlands/1/2020 H5N1. For each branch, bootstrap values (1000 replicates) are shown in percentages. The branches corresponding to discrete genotypes are labelled as AB, AF, CH and BB and coloured. The expanded version of the tree is shown in Supplementary Figure S1. In (**B**), the schematic representation of the segment composition of the AB, AF, CH and BB genotypes is presented [35].

Phylogenetic analysis of the H5 sequences suggested that the CZE 2022/2023 H5N1 strains belonged to the 2.3.4.4b H5 lineage. The concatenated phylogenetic tree showed that the H5N1 genomes could be assigned to four different genotypes, which were named AB, AF, CH and BB according to the recent classification system [35] (Figure 2A). Genotypes AF and BB form two distant clusters, reflecting their distinct origin through reassortment with different low-pathogenicity viruses. Conversely, the relationship between genotypes AB and CH is much closer, with genotype CH being derived from AB-like viruses through an exchange of the NP segment [35] (Figure 2B). The concatenated genomic tree further suggested that, within genotype AB, the Czech H5N1 2022/2023 strains were separated into three well-supported subclades. The observed genotype structure is fully consistent with trees calculated individually for each genomic segment (Supplementary Figures S2–S9).

### 3.3. Molecular Epizootology of the Czech 2022/2023 H5N1 Viruses

**Figure 3:**
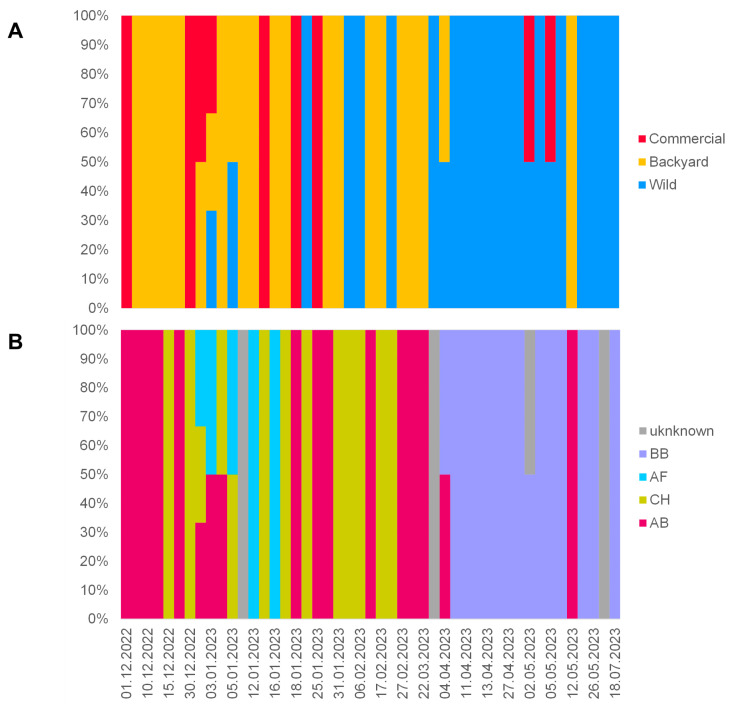
Histograms showing the time course of H5N1 virus detections in the Czech Republic during the 2022/2023 season, categorized using bird categories (**A**), together with the associated genotypes (**B**).

Figure 3A shows the timeline scheme of virus detections during the 2022/2023 H5N1 season in the particular bird category (wild, backyard and commercial) along with the assigned genotypes. As can be seen, from December 2022 to April 2023, the disease was mainly detected in the backyard and commercial poultry farms distributed throughout the country, with a very sporadic identification in wild birds. Nevertheless, where available, the viruses from wild birds show very close relationships to poultry (Supplementary Figure S1).

However, the pattern of the data has changed dramatically since the beginning of April 2023, with a massive outbreak in black-headed gulls. This epizootic event also changed the frequency of the genotypes detected (Figure 3B). Until April 2023, the season was dominated by two genotypes, AB and CH, with a short episode of genotype AF. Most outbreaks in commercial poultry were caused by genotypes AB and CH, while three genotypes, AB, CH and AF, were detected in backyard poultry. However, from April 2023, these were replaced by genotype BB, which was massively imported via black-headed gulls. However, at least the AB genotype appears to have remained in circulation, as indicated by its detection in backyard poultry at the end of the season (IDs 7531/23; 12 May 2023). All genotypes were distributed throughout the Czech Republic, with an interesting spatio-temporal cluster in the northeastern part where three H5N1 genotypes, AB, AF and CH, were detected within one month (28 December 2022–20 January 2023; Figure 1).

Despite the wide distribution of genotype BB, only one transmission event from gulls to poultry was recorded, represented by a commercial turkey farm (IDs 6734/23, 7123/23, 7124/23). Phylogenetic analysis showed that the H5N1 strains from turkeys were closely related by all genomic segments to the Polish H5N1 strains A/black_headed_gull/Poland/MB143-M1/2023 and A/black_headed_gull/Poland/MB168-M2/2023 (Supplementary Figures S2–S9), which were collected shortly before or during the course of the outbreak in turkeys. These strains also shared the minor cleavage site motif mentioned above, further emphasizing their common origin. Given the widespread distribution of the BB genotype, several infected common tern and peregrine falcon carcasses were found as a result of habitat sharing or hunting or scavenging of the infected gulls.

**Figure 4:**
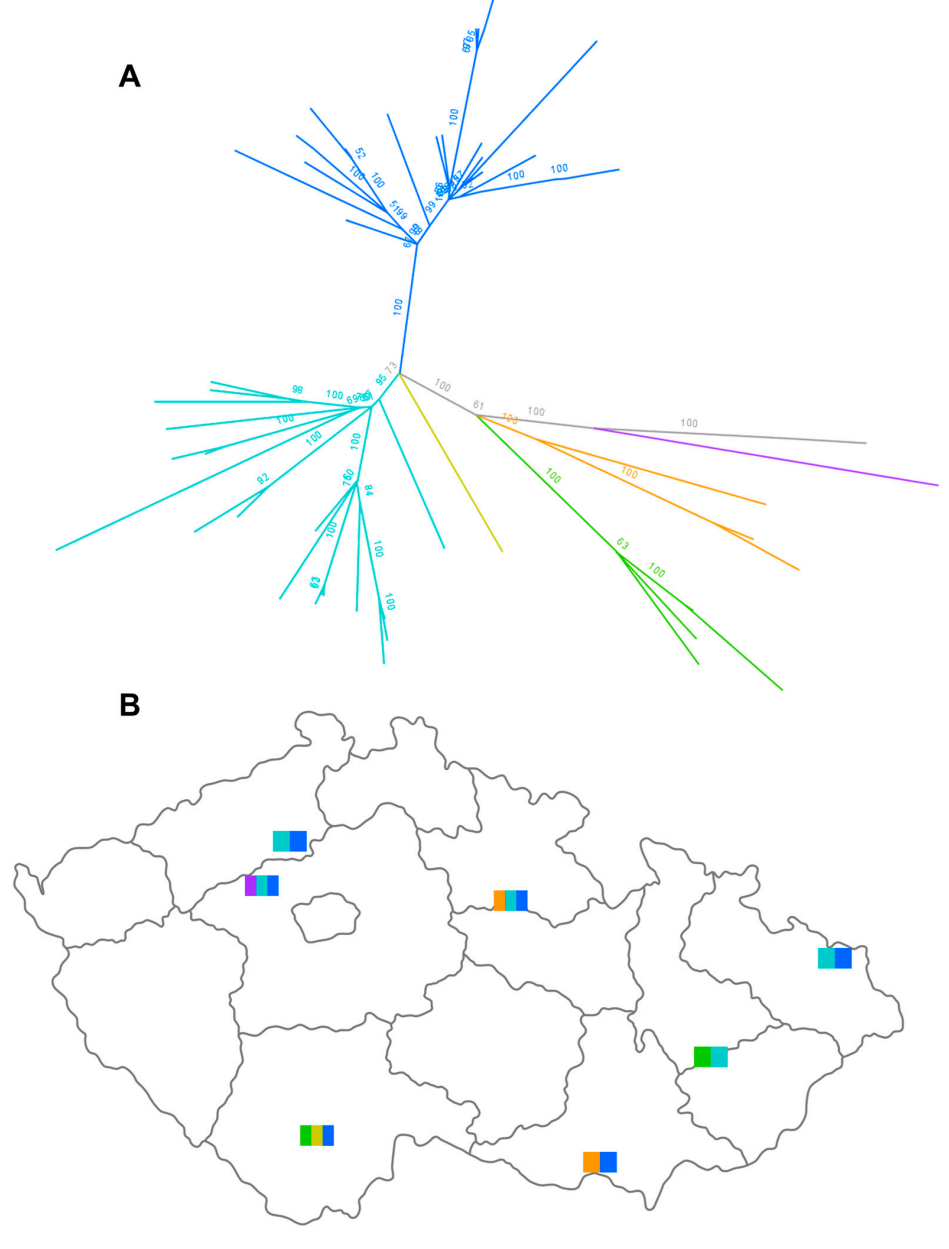
Local diversity of genotype BB H5N1 viruses detected in gulls in the Czech Republic. In (**A**), the discrete phylogenetic lineages of genotype BB in a concatenated genomic sub-tree are indicated using different colours. The prototype strain, A/Herring_gull/France/22P015977/2022 H5N1, is highlighted in grey. A detailed tree with expanded IDs is provided in Supplementary Figure S10. In (**B**), sites with distinct strains of genotype BB are shown. Flag colours correspond to (**A**).

To further explore the genetic diversity within the BB genotype, we attempted to sequence H5N1 strains from all individual gulls and terns collected at the same time and place. Thus, data are available from up to seven birds from the same site (Supplementary Table S1). As a result, the subtree of concatenated BB genomes (Figure 4) revealed a dispersion of the viruses into several highly supported subclades, even those collected at the same site. For example, IDs 5487/23-2, 4 and 5, comprising three H5N1 strains from three different birds collected at the same site, were placed into three divergent subclades. A similar pattern was also observed in the individual segment trees. This suggests a high local diversity of the H5N1 strains detected in gulls and terns.

**Figure 5:**
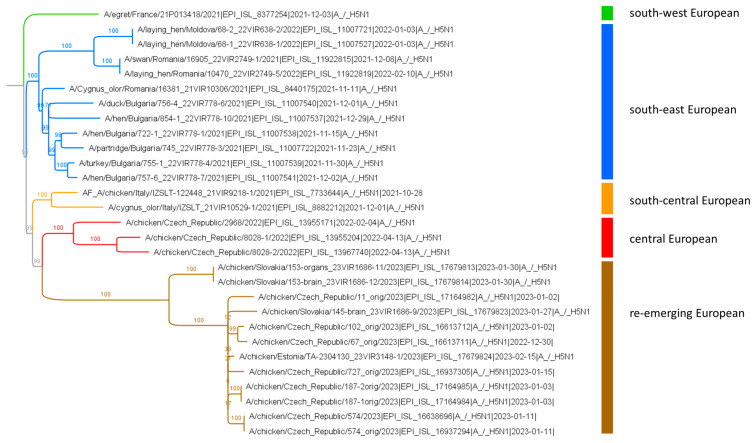
A concatenated genomic tree of the H5N1 viruses belonging to the AF genotype detected in Europe during the 2021/2022 and 2022/2023 seasons. The discrete phylogenetic lineages are indicated using different colours.

Another interesting finding of the 2022/2023 season was the reappearance of genotype AF. The prototype status for this genotype was assigned to an H5N1 virus detected in Italy at the beginning of the 2021/2022 season. Based on the available sequence data, the AF genotype appeared to be widespread in the previous season, mainly throughout southern Europe. Phylogenetic analysis revealed four distinct AF genotype subclades, which for the purpose of this study can be described as follows: (i) south-eastern (Bulgaria, Romania and Moldova); (ii) south-central (Italy); (iii) south-western (France) and (iv) central European (Czech Republic). However, during the 2022/2023 season, novel AF strains appeared throughout the Czech Republic in several backyard poultry farms located hundreds of kilometres apart (Figure 3). This is consistent with the available genomes in the GISAID database, also found in poultry in Slovakia and Estonia, (Figure 5) and suggests a wider geographical distribution. The genomic tree showed that all re-emerging AF strains form a separate cluster with the closest relationship to the AF viruses detected in the Czech Republic during the previous HPAI season.

## 4. Discussion

The 2022/2023 H5N1 HPAI season was the most serious for the poultry industry ever recorded in the Czech Republic. Despite the number of outbreaks in poultry being roughly comparable to the previous seasons [36,37], for the first time, the number of culled birds approached one million. This was circa three times higher than in previous years and was mainly due to an outbreak in a single poultry farm of ~750,000 laying hens. Infection of the critical farm presented a great challenge since an unimaginable number of animals and eggs had to be destroyed. This particular outbreak management, including disinfection, required a coordinated effort of around 120 people over 10 days and generated more than 1000 tons of contaminated waste, which posed another challenge for disposal.

In general, the outbreaks in Czech commercial poultry followed the same scenario as observed in previous years [36,37] and reported elsewhere in Europe [38,39,40]. The disease has always appeared suddenly, even in commercial poultry farms with a high level of biosecurity [41]. In commercial poultry, the virus then spreads rapidly to other breeding compartments and sheds with no possibility of effective interruption. Available data suggest that virus particles are ubiquitous in dust on various surfaces at the affected farms [42]. Therefore, the environment may be an important source of contamination for neighbouring sheds. This was also supported by our observation that all of the surface swabs taken from an infected flock were positive for the H5N1 HPAI virus. However, the detailed routes of transmission and the mechanisms of further spread remain largely unknown. The only effective measure was the depopulation of the affected farms and subsequent sanitation. A critical ecological aspect of many poultry farms is their proximity to water bodies or wetlands [43], which greatly increases the risk of virus transmission from wild waterfowl. This factor was also present in the above highly populated poultry farm, which was located close to an important ornithological site.

In addition to the critical location, the shift in the ecological behaviour of the H5N1 HPAI virus observed since 2021 [6], which has intensified in the last two seasons, has not only changed the infection dynamics in European wild birds but also has a direct impact on poultry infections. The resident HPAI strains persisting in wild birds from one season to the next may significantly contribute to outbreaks in poultry. This has been suggested at the local level using the reappearance of genotype AF in domestic poultry in the Czech Republic. In addition, this genotype showed a much wider geographical distribution compared to the previous season [36]. Similarly, but from a broader perspective, enzootic circulation is clearly evident, at least for the genotype BB, which was first identified in May 2022 and has since persisted in colony-breeding seabirds in northern Europe [17]. From December 2022 onwards, genotype BB began to spread massively inland [17] and was responsible for an outbreak in Czech commercial turkeys.

The observed genotype diversity among the Czech H5N1 viruses suggests the importance of large-scale genomic sequencing during the HPAI season. In the absence of a comprehensive surveillance programme in wild birds, we tried to obtain genomic information from as many samples as possible, even from the same site and sampling occasion. We also included samples with high Cq values, which required a lot of effort and a fine-tuned and sensitive protocol. Using this approach, we were able to obtain sequence information from 94.5% of outbreaks and sampling sites. This “highly sensitive molecular surveillance (HSMS)” approach was instrumental in revealing the AF genotype, which would otherwise remain largely undetected, as it represents only ~9% of all outbreaks. In addition, it was detected only in backyard poultry, highlighting major gaps in our knowledge of HPAI circulation in wild birds.

Given the background level of circulation, the origin of the AF genotype in the 2022/2023 season is unclear. The phylogenetic tree revealed a sister clade relationship with the Czech AF viruses from the previous season. We hypothesize that the common ancestor of both clades persisted in the local wild bird population without reassortment and seeded the AF viruses in the next season. This is further supported by the temporal sequence of detection of AF viruses and their relatively high frequency (19%) between the outbreaks in Czech backyard poultry.

The HSMS approach also revealed an interesting focus in the northeast of the Czech Republic, where all four H5N1 genotypes were detected in a relatively small geographical area and where, given the time frame of sampling, at least the AB, AF and CH genotypes must have been cocirculated. However, no intrasubtype reassortants were found among these viruses.

The unprecedented mass mortality in gulls also provided a unique opportunity to study the H5N1 HPAI virus in this wild bird species. From this perspective, another interesting finding of HSMS was a relatively high local diversity of genotype BB viruses in gulls and apparently also in terns. Phylogenetic analysis has suggested that individual gull strains obtained from the same area were distributed into distinct subclades and, conversely, that distinct subclades included viruses collected from different birds in the same location. This variability was observed in the samples collected from seven out of twelve geographically separated outbreaks and appeared to be generalisable to all sites of massive gull mortality. Furthermore, given only the fraction of dead birds sampled, the local H5N1 diversity must have been even higher than observed. This adds further depth to the previous observations on the diversity of avian viruses at the local scale [44,45,46,47], down to the level of strains.

The observed genetic diversity in gulls can be attributed to the length of time the virus has been present in the European gull population, together with the geographical spread and complex multidirectional migratory behaviour. Gulls are considered a species that moves the avian influenza virus faster than any other avian host [48]. Available data suggest that the Czech population of black-headed gulls overwinters in many European regions, reaching the North Sea coast in the Netherlands and Belgium and the Atlantic coasts of France and the British Isles in the northwest. Another population migrates to the alpine lakes in Switzerland and spreads southwestwards towards the Mediterranean Sea, reaching southern France, Spain, Portugal, Algeria and Morocco. A small population of Czech gulls also moves southeastwards into the Balkan countries and onto Italy and Greece. About 63% of the birds stay within 1000 km of the nesting site, and ~11% migrate even further. From the spring of the following year, gulls from all these destinations return to nesting sites in central Europe [49], including the Czech Republic, bringing phylogenetically different strains of H5N1 even to the same nesting site.

The emergence of SARS-CoV-2 clearly demonstrated the importance of real-time molecular surveillance to track its spread and detect mutations that could potentially affect pathogenicity, transmission and countermeasures. However, this could only have been achieved through the timely sharing of sequences and associated metadata. Unfortunately, this effort has not yet been fully applied to the molecular surveillance of H5Nx HPAI in Europe, and considering the significance and impact of the last pan-European infection wave, the sequence database contained only a few H5N1 sequences for most of the time of the outbreak. Only timely data sharing can effectively contribute to tracking the spread and detecting molecular changes that influence the infectivity of this potentially zoonotic pathogen.

## Data Availability

All sequences generated in this study have been uploaded to the Global Initiative on Sharing All Influenza Data (GISAID) EpiFlu database, with accession numbers listed in Supplementary Table S1. All sequence analyses are included in the manuscripts or in the Supplementary Files and Figures. All interactive maps related to Figure 1 are available at https://www.google.com/maps/d/u/1/edit?mid=1F2miwu6f3LwcERhDkZGK-ztunikfBg0&ll=49.757942391979654%2C15.579510149999987&z and https://www.google.com/maps/d/viewer?mid=1TK7NNdEvvBuOskqEvAHU0KMn6RLhGAc&ll=49.929839528405346%2C15.65820290000002&z.

## Supplementary Materials

The following supporting information can be downloaded at: https://www.mdpi.com/article/10.3390/v16020221/s1, Supplementary Table S1. Overview of all H5N1 outbreaks and sampling events in chronological order. Supplementary Figure S1. The expanded version of Figure 2A. The H5N1 virus names were coloured according to the bird categories: red—commercial poultry, orange—backyard poultry, blue—wild birds and green—birds of prey. Supplementary Figures S2–S9. Phylogenetic analysis of Czech 2022/2023 H5N1 strains in the context of available European H5N1 genomes. The ML tree (best-fit substitution models according to Bayesian information criterion: PB2, PB1, PA: UNREST+FO+I+G4; H5, NP: GTR+F+I+G4; N1: UNREST+FO+G4; MP: TVMe+I+G4; NS: TVM+F+I+G4) was calculated separately for each genomic segment based on European H5N1 sequences collected between September 2022 and June 2023, including the reference and master strains, and stored in the GISAID EpiFlu database. PB2 n = 1342, PB1 n = 1335, PA = 1348, H5 n = 1425, NP n = 1340, N1 n = 1376, MP n = 1377 and NS n = 1343. The names of the Czech 2022/2023 H5N1 strains were coloured according to the bird categories: red—commercial poultry, orange—backyard poultry, blue—wild birds and green—birds of prey. For each branch, the bootstrap values (1000 replicates) are given in percentages. All trees except N1 were rooted to A/chicken/Scotland/1959 H5N1. The N1 tree was rooted to A/goose/Guangdong/1/1996. The branches of the tree corresponding to discrete genotypes were highlighted according to Figure 2B. Only the subtrees of interest are shown for certain trees. Supplementary Figure S10. An extended view of Figure 4A.
