# Supplementary material for: Enzootic Circulation, Massive Gull Mortality and Poultry Outbreaks during the 2022/2023 High-Pathogenicity Avian Influenza H5N1 Season in the Czech Republic
Source: Viruses. 2024 Jan 31;16(2):221. doi: 10.3390/v16020221 (PMC10892573; doi:10.3390/v16020221)
Supplement: Supplementary file 1 [file viruses-16-00221-s001.zip › SupplementaryTable 1.pdf]

Supplementary Table 1. The overview of all H5N1 outbreaks and sampling events listed in chronological order.

Strain ID	Collection Date	Category[#]	Name	Accession no.	Genotype
22394	2022-12-01	Commercial A	A/domestic_duck/Czech_Reublic/22394/2022	EPI_ISL_17584807	AB
22910	2022-12-08	Backyard	A/chicken/Czech_Republic/22910-1/2022<br>A/chicken/Czech_Republic/22910-2/2022<br>A/chicken/Czech_Republic/22910-3/2022	EPI_ISL_16638641<br>EPI_ISL_16638642<br>EPI_ISL_16638643	AB
22911	2022-12-08	Backyard	A/chicken/Czech_Republic/22911-1/2022	EPI_ISL_16638676	AB
22912	2022-12-08	Backyard	A/domestic_duck/Czech_Republic/22912-1/2022	EPI_ISL_16638657	AB
22968	2022-12-10	Backyard	A/chicken/Czech_Republic/22968-3/2022<br>A/chicken/Czech_Republic/22968-2/2022<br>A/chicken/Czech_Republic/22968-1/2022<br>A/chicken/Czech_Republic/22968_orig/2022	EPI_ISL_16937302<br>EPI_ISL_16937303<br>EPI_ISL_16937304<br>EPI_ISL_16997293	AB
22969	2022-12-11	Backyard	A/chicken/Czech_Republic/22969-1/2022<br>A/chicken/Czech_Republic/22969-2/2022<br>A/chicken/Czech_Republic/22969-4/2022<br>A/chicken/Czech_Republic/22969-5/2022	EPI_ISL_16937298<br>EPI_ISL_16937299<br>EPI_ISL_16937300<br>EPI_ISL_16937301	AB
23309	2022-12-15	Backyard	A/chicken/Czech_Republic/23309-1orig/2022<br>A/chicken/Czech_Republic/23309-2orig/2022<br>A/chicken/Czech_Republic/23309-4orig/2022	EPI_ISL_17164986<br>EPI_ISL_17164987<br>EPI_ISL_17164988	CH
23869	2022-12-28	Backyard	A/chicken/Czech_Republic/23869-1orig/2022<br>A/chicken/Czech_Republic/23869-2orig/2022	EPI_ISL_17164989<br>EPI_ISL_17164990	AB
24009	2022-12-30	Commercial B	A/chicken/Czech_Republic/24009-1/2022<br>A/chicken/Czech_Republic/24009-2/2022	EPI_ISL_16937296<br>EPI_ISL_16937297	CH
11	2023-01-02	Backyard	A/chicken/Czech_Republic/11_orig/2023	EPI_ISL_17164982	AF
58	2023-01-02	Commercial B	A/chicken/Czech_Republic/58-shed-2A_orig/2023	EPI_ISL_17164991	CH
66	2023-01-02	Commercial C	A/turkey/Czech_Republic/66_orig/2023	EPI_ISL_17164992	AB
67	2023-01-03	Backyard	A/chicken/Czech_Republic/67_orig/2023	EPI_ISL_16613711	AF
94	2023-01-03	Wild	A/greylag_goose/Czech_Republic/94_orig/2023	EPI_ISL_17164995	AB
102	2023-01-03	Backyard	A/chicken/Czech_Republic/102_orig/2023	EPI_ISL_16613712	AF
122	2023-01-04	Commercial B	unknown	-	-
178	2023-01-04	Commercial B	A/chicken/Czech_Republic/178-shed-1A_orig/2023	EPI_ISL_16613713	CH
179	2023-01-04	Commercial B	A/chicken/Czech_Republic/179-shed-1B_orig/2023<br>A/chicken/Czech_Republic/179/2023	EPI_ISL_16613714<br>EPI_ISL_16937292	CH
180	2023-01-04	Commercial B	A/chicken/Czech_Republic/180-shed-3A_orig/2023<br>A/chicken/Czech_Republic/180/2023	EPI_ISL_16613715<br>EPI_ISL_16937293	CH
181	2023-01-04	Commercial B	A/chicken/Czech_Republic/181-shed-3B_orig/2023	EPI_ISL_17164983	CH
185	2023-01-04	Backyard	A/chicken/Czech_Republic/185_orig/2023	EPI_ISL_16613716	AB
186	2023-01-05	Wild	A/mallard/Czech_Republic/186_orig/2023	EPI_ISL_16613717	CH
187	2023-01-05	Backyard	A/chicken/Czech_Republic/187-1orig/2023<br>A/chicken/Czech_Republic/187-2orig/2023	EPI_ISL_17164984<br>EPI_ISL_17164985	AF
327	2023-01-06	Backyard	unknown	-	
574	2023-01-12	Backyard	A/chicken/Czech_Republic/574/2023<br>A/chicken/Czech_Republic/574_orig/2023	EPI_ISL_16638696<br>EPI_ISL_16937294	AF
636	2023-01-13	Commercial D	A/domestic_duck/Czech_Republic/636_orig/2023	EPI_ISL_16937295	CH
872	2023-01-13	Commercial B	A/chicken/Czech_Republic/872_orig/2023	EPI_ISL_16939160	CH
727	2023-01-16	Backyard	A/chicken/Czech_Republic/727_orig/2023	EPI_ISL_16937305	AF
811	2023-01-17	Backyard	A/swan/Czech_Republic/811-2/2023	EPI_ISL_16937306	CH
951	2023-01-18	Commercial E	A/domestic_duck/Czech_Republic/951_orig/2023	EPI_ISL_16937307	AB
1220	2023-01-20	Commercial E	A/environment/Czech_Republic/1220-3_hall-8/2023<br>A/environment/Czech_Republic/1220-9_hall-8/2023<br>A/environment/Czech_Republic/1220-11_hall-8/2023	EPI_ISL_16937309<br>EPI_ISL_16937310<br>EPI_ISL_16937311	AB

1021	2023-01-20	Wild	A/mallard/Czech_Republic/1021/2023	EPI_ISL_16937308	CH
1398	2023-01-25	Commercial F	A/domestic_duck/Czech_Republic/1398/2023	EPI_ISL_16997751	AB
1600	2023-01-29	Backyard	A/guineafowl/Czech_Republic/1600-1/2023<br>A/guineafowl/Czech_Republic/1600-2/2023<br>A/chicken/Czech_Republic/1600-1/2023<br>A/chicken/Czech_Republic/1600-2/2023	EPI_ISL_16997754<br>EPI_ISL_16997755<br>EPI_ISL_16997756<br>EPI_ISL_16997757	AB
1690	2023-01-31	Backyard	A/chicken/Czech_Republic/1690/2023<br>A/chicken/Czech_Republic/1690_orig/2023	EPI_ISL_16997752<br>EPI_ISL_16997753	CH
1792	2023-02-02	Wild	A/mallard/Czech_Republic/1792_orig/2023	EPI_ISL_16997758	CH
2022	2023-02-06	Wild	A/swan/Czech_Republic/2022/2023	EPI_ISL_16997759	CH
2749	2023-02-16	Backyard	A/chicken/Czech_Republic/2749-1/2023<br>A/chicken/Czech_Republic/2749-2/2023<br>A/chicken/Czech_Republic/2749-3/2023<br>A/domestic_duck/Czech_Republic/2749-5/2023	EPI_ISL_17015366<br>EPI_ISL_17015367<br>EPI_ISL_17015368<br>EPI_ISL_17015369	AB
2750	2023-02-16	Backyard	A/chicken/Czech_Republic/2750-1/2023<br>A/chicken/Czech_Republic/2750-2/2023<br>A/chicken/Czech_Republic/2750-3/2023<br>A/chicken/Czech_Republic/2750-4/2023<br>A/chicken/Czech_Republic/2750-5/2023	EPI_ISL_17015359<br>EPI_ISL_17015360<br>EPI_ISL_17015362<br>EPI_ISL_17015363<br>EPI_ISL_17015364	AB
2753	2023-02-17	Backyard	A/domestic_goose/Czech_Republic/2753/2023	EPI_ISL_17015365	CH
3051	2023-02-23	Wild	A/great_egret/Czech_Republic/3051-1orig/2023<br>A/great_egret/Czech_Republic/3051-2orig/2023	EPI_ISL_17164993<br>EPI_ISL_17164994	CH
3192	2023-02-27	Backyard	A/chicken/Czech_Republic/3192_orig/2023	EPI_ISL_17470101	AB
3446	2023-03-02	Backyard	A/chicken/Czech_Republic/3446/2023<br>A/chicken/Czech_Republic/3446_orig/2023	EPI_ISL_17470103<br>EPI_ISL_17470102	AB
4463	2023-03-22	Backyard	A/chicken/Czech_Republic/4463_orig/2023	EPI_ISL_17470104	AB
4570	2023-03-24	Wild	unknown	-	
5152	2023-04-04	Wild	A/black-headed_gull/Czech_Republic/5152-1/2023<br>A/black-headed_gull/Czech_Republic/5152-2/2023<br>A/black-headed_gull/Czech_Republic/5152-3/2023<br>A/black-headed_gull/Czech_Republic/5152-4/2023<br>A/black-headed_gull/Czech_Republic/5152-5/2023	EPI_ISL_17470105<br>EPI_ISL_17470106<br>EPI_ISL_17470107<br>EPI_ISL_17470108<br>EPI_ISL_17470109	BB
5215	2023-04-04	Backyard	A/chicken/Czech_Reublic/5215_orig/2023	EPI_ISL_17584809	AB
5295	2023-04-05	Wild	A/black-headed_gull/Czech_Republic/5295-3/2023	EPI_ISL_17584810	BB
5425	2023-04-11	Wild	A/black-headed_gull/Czech_Republic/5425-1/2023<br>A/black-headed_gull/Czech_Republic/5425-3/2023	EPI_ISL_17584811<br>EPI_ISL_17584812	BB
5452	2023-04-11	Wild	A/black-headed_gull/Czech_Republic/5452-1/2023<br>A/black-headed_gull/Czech_Republic/5452-2/2023<br>A/black-headed_gull/Czech_Republic/5452-3/2023<br>A/black-headed_gull/Czech_Republic/5452-4/2023	EPI_ISL_17584813<br>EPI_ISL_17584814<br>EPI_ISL_17584815<br>EPI_ISL_17584816	BB
5487	2023-04-12	Wild	A/black-headed_gull/Czech_Republic/5487-2/2023<br>A/black-headed_gull/Czech_Republic/5487-4/2023<br>A/black-headed_gull/Czech_Republic/5487-5/2023	EPI_ISL_17584817<br>EPI_ISL_17584818<br>EPI_ISL_17584819	BB
5695	2023-04-13	Wild	A/black-headed_gull/Czech_Republic/5695-1/2023<br>A/black-headed_gull/Czech_Republic/5695-2/2023<br>A/black-headed_gull/Czech_Republic/5695-3/2023<br>A/black-headed_gull/Czech_Republic/5695-4/2023<br>A/black-headed_gull/Czech_Republic/5695-5/2023<br>A/black-headed_gull/Czech_Republic/5695-7/2023<br>A/black-headed_gull/Czech_Republic/5695-8/2023	EPI_ISL_17584820<br>EPI_ISL_17584821<br>EPI_ISL_17584822<br>EPI_ISL_17584823<br>EPI_ISL_17584824<br>EPI_ISL_17584825<br>EPI_ISL_17584826	BB
6066	2023-04-20	Wild	A/black-headed_gull/Czech_Republic/6066-5/2023	EPI_ISL_17584827	BB
6577	2023-04-27	Wild	A/black-headed_gull/Czech_Republic/6577-1/2023<br>A/black-headed_gull/Czech_Republic/6577-2/2023<br>A/black-headed_gull/Czech_Republic/6577-3/2023<br>A/black-headed_gull/Czech_Republic/6577-4/2023<br>A/black-headed_gull/Czech_Republic/6577-5/2023	EPI_ISL_17716810<br>EPI_ISL_17716811<br>EPI_ISL_17716812<br>EPI_ISL_17716813<br>EPI_ISL_17650916	BB
6633	2023-04-28	Wild	A/black-headed_gull/Czech_Republic/6633-1/2023<br>A/black-headed_gull/Czech_Republic/6633-2/2023	EPI_ISL_17716814<br>EPI_ISL_17716815	BB

6634	2023-04-28	Wild	A/black-headed_gull/Czech_Republic/6634-1/2023<br>A/black-headed_gull/Czech_Republic/6634-2/2023<br>A/black-headed_gull/Czech_Republic/6634-3/2023<br>A/black-headed_gull/Czech_Republic/6634-4/2023<br>A/black-headed_gull/Czech_Republic/6634-5/2023	EPI_ISL_17716816<br>EPI_ISL_17650917<br>EPI_ISL_17650918<br>EPI_ISL_17650919<br>EPI_ISL_17650920	BB
6734	2023-05-02	Commercial G	A/turkey/Czech_Republic/6734_orig/2023	EPI_ISL_17650921	BB
6735	2023-05-02	Wild	unknown	-	-
6947	2023-05-04	Wild	A/black-headed_gull/Czech_Republic/6947-1/2023<br>A/black-headed_gull/Czech_Republic/6947-3/2023<br>A/black-headed_gull/Czech_Republic/6947-4/2023	EPI_ISL_17716817<br>EPI_ISL_17716818<br>EPI_ISL_17716819	BB
7123	2023-05-05	Commercial G	A/turkey/Czech_Republic/7123-1/2023<br>A/turkey/Czech_Republic/7123-2/2023	EPI_ISL_17716820<br>EPI_ISL_17716821	BB
7124	2023-05-05	Commercial G	A/turkey/Czech_Republic/7124-1/2023<br>A/turkey/Czech_Republic/7124-2/2023	EPI_ISL_17716822<br>EPI_ISL_17716823	BB
8807	2023-05-05	Wild	A/common_tern/Czech_Republic/8807-1/2023<br>A/common_tern/Czech_Republic/8807-4/2023<br>A/common_tern/Czech_Republic/8807-5/2023	EPI_ISL_17884626<br>EPI_ISL_17884627<br>EPI_ISL_17884628	BB
7334	2023-05-11	Wild	A/black-headed_gull/Czech_Republic/7334-4/2023<br>A/black-headed_gull/Czech_Republic/7334-5/2023	EPI_ISL_17716824<br>EPI_ISL_17716825	BB
7459	2023-05-11	Wild	A/peregrine_falcon/Czech_Republic/7459-1/2023<br>A/peregrine_falcon/Czech_Republic/7459-2/2023<br>A/peregrine_falcon/Czech_Republic/7459-3/2023	EPI_ISL_17716826<br>EPI_ISL_17716827<br>EPI_ISL_17716828	BB
7531	2023-05-12	Backyard	A/chicken/Czech_Republic/7531-1orig/2023<br>A/chicken/Czech_Republic/7531-1/2023<br>A/chicken/Czech_Republic/7531-2/2023<br>A/guinea_fowl/Czech_Republic/7531-2orig/2023<br>A/chicken/Czech_Republic/7531-3/2023<br>A/guinea_fowl/Czech_Republic/7531-4/2023<br>A/guinea_fowl/Czech_Republic/7531-5/2023	EPI_ISL_17716832<br>EPI_ISL_17716829<br>EPI_ISL_17716830<br>EPI_ISL_17716809<br>EPI_ISL_17716831<br>EPI_ISL_17716807<br>EPI_ISL_17716808	AB
7735	2023-05-17	Wild	A/peregrine_falcon/Czech_Republic/7735-1/2023<br>A/peregrine_falcon/Czech_Republic/7735-2/2023<br>A/peregrine_falcon/Czech_Republic/7735-2_chymus/2023	EPI_ISL_17732338<br>EPI_ISL_17732339<br>EPI_ISL_17732340	BB
8420	2023-05-26	Wild	A/peregrine_falcon/Czech_Republic/8420/2023	EPI_ISL_17884625	BB
8527	2023-05-30	Wild	unknown	-	-
10854	2023-07-18	Wild	A/peregrine falcon/Czech Republic/10854/2023	EPI_ISL_18048779	BB

Capital letters denote multiple sampling of the same farm.
