# Supplementary figures and images for: Enzootic Circulation, Massive Gull Mortality and Poultry Outbreaks during the 2022/2023 High-Pathogenicity Avian Influenza H5N1 Season in the Czech Republic
Source: Viruses. 2024 Jan 31;16(2):221. doi: 10.3390/v16020221 (PMC10892573; doi:10.3390/v16020221)

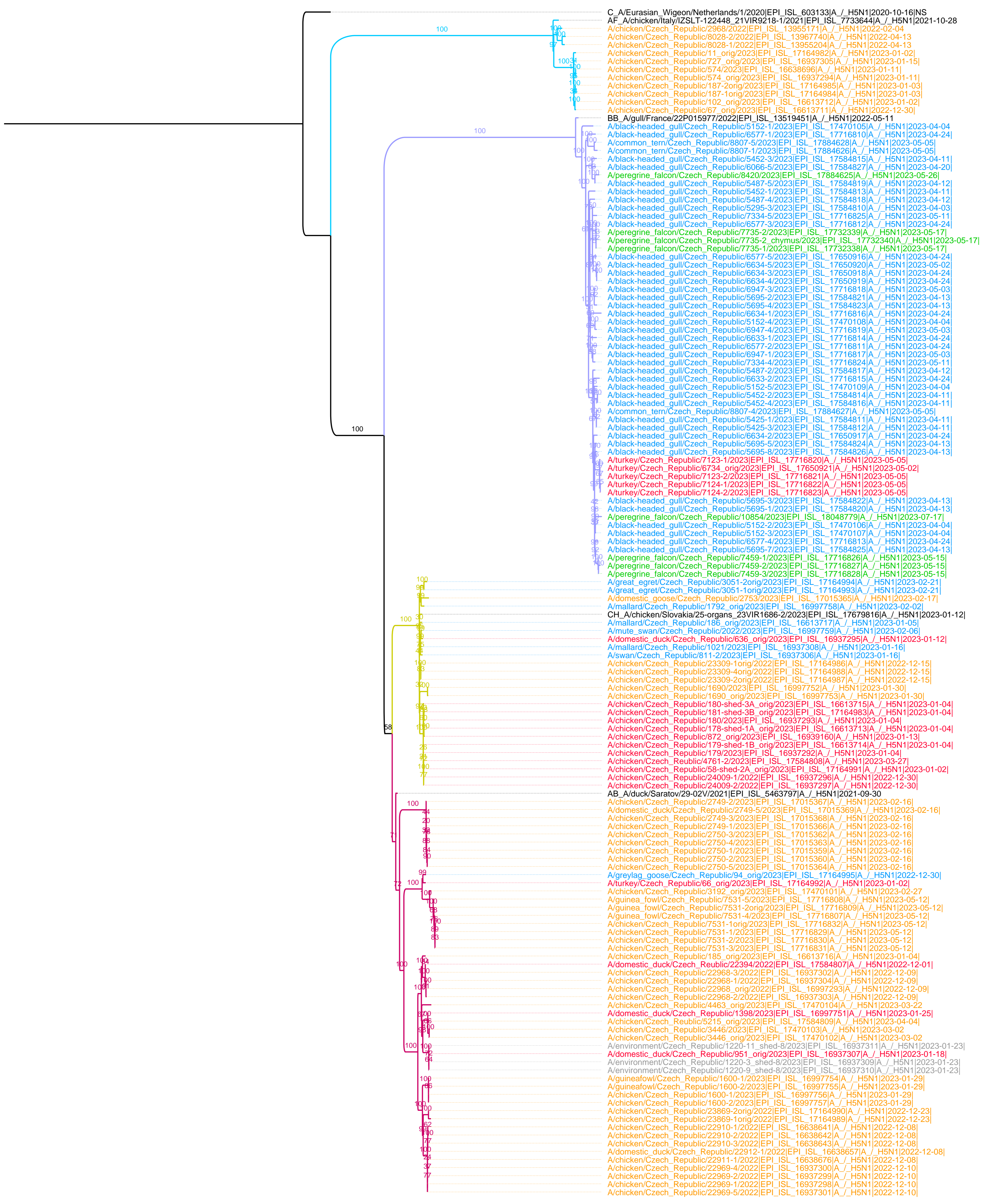

Supplement: Supplementary file 1 [file viruses-16-00221-s001.zip › Supplementary Figure S1.pdf]

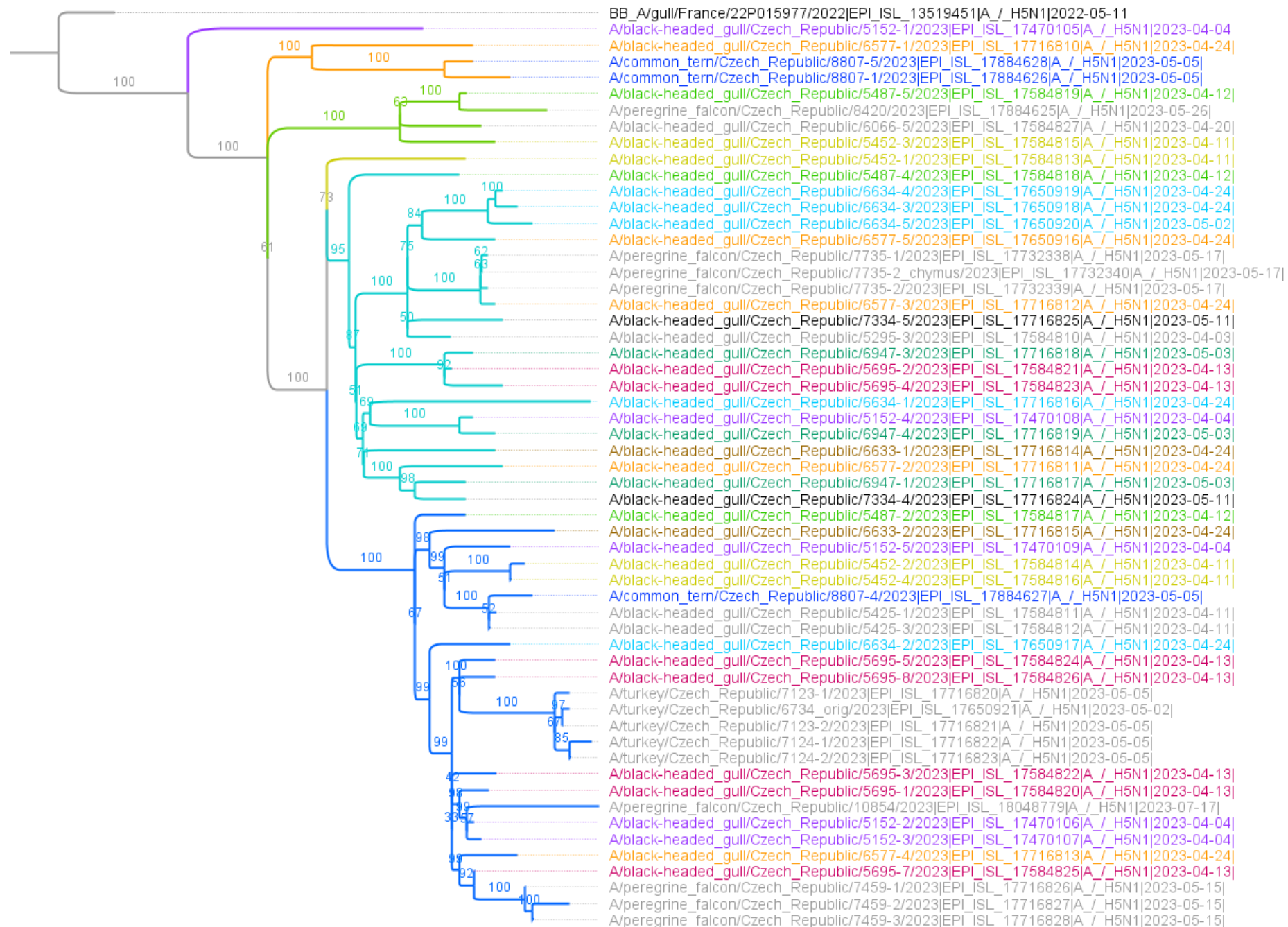

Supplement: Supplementary file 1 [file viruses-16-00221-s001.zip › Supplementary Figure S10.pdf]

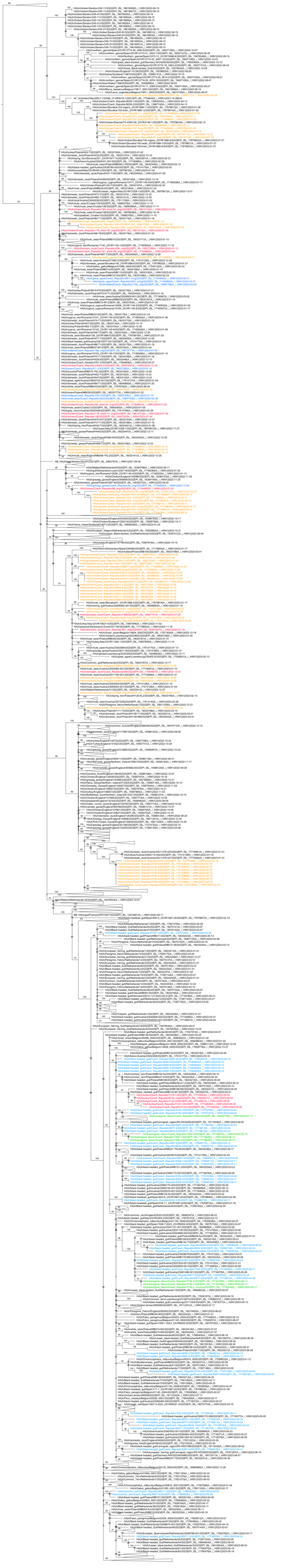

Supplement: Supplementary file 1 [file viruses-16-00221-s001.zip › Supplementary Figure S5-H5.pdf]
