# Supplementary material for: Enzootic Circulation, Massive Gull Mortality and Poultry Outbreaks during the 2022/2023 High-Pathogenicity Avian Influenza H5N1 Season in the Czech Republic
Source: Viruses. 2024 Jan 31;16(2):221. doi: 10.3390/v16020221 (PMC10892573; doi:10.3390/v16020221)

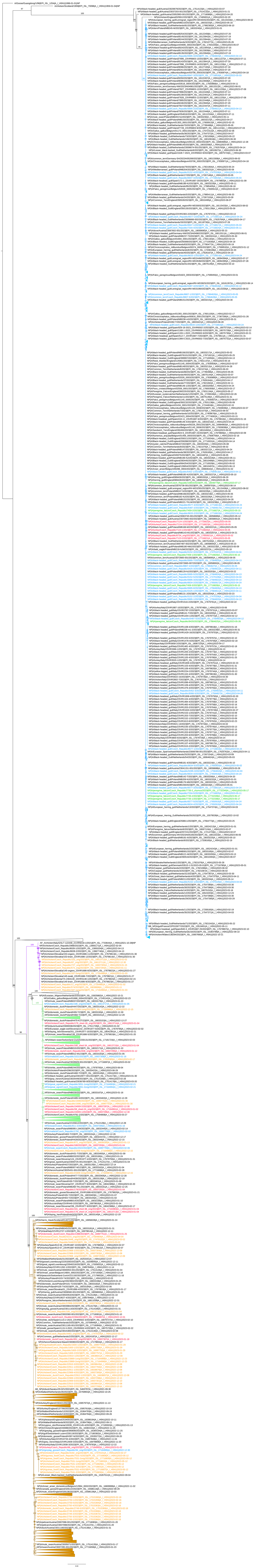

Supplement: Supplementary file 1 [file Supplementary Figure S6-NP.pdf › Supplementary Figure S6-NP.pdf]
